# 2171. Clinical Characteristics and Outcomes of Immunocompromised Patients with Enterococcal Pneumonia

**DOI:** 10.1093/ofid/ofac492.1791

**Published:** 2022-12-15

**Authors:** Leama Ajaka, Shandra R Day, Courtney Hebert, Nora Colburn, Christina Liscynesky

**Affiliations:** Ohio State University Wexner Medical Center, Columbus, Ohio; Ohio State University Wexner Medical Center, Columbus, Ohio; The Ohio State University, Columbus, Ohio; The Ohio State University Wexner Medical Center, Columbus, Ohio; The Ohio State Univiersity Wexner Medical Center, Columbus, Ohio

## Abstract

**Background:**

Although *Enterococcus* species can colonize and cause infection in multiple body sites, it is considered a rare cause of pneumonia (PNA). The study objective was to describe clinical outcomes in immunocompromised patients with PNA and *Enterococcus* in respiratory cultures to better elucidate the pathogenic role played by *Enterococci*.

**Methods:**

A retrospective, descriptive observational study was conducted on immunocompromised patients with *Enterococcus* spp on lower respiratory culture from January 1, 2015 to September 22, 2021 with clinical PNA. Data was collected on patient demographics, microbiologic details including bacteremia, other potential infectious causes of PNA and clinical outcomes.

**Results:**

116 cases of immunocompromised patients with PNA and *Enterococcus* spp on respiratory culture were identified. Two patients also grew *Enterococcus* spp on pleural fluid and 1 on lung tissue. Vancomycin-resistant *Enterococcus* grew in 41/116 (35%) cases. Six (5%) patients had a bloodstream infection due to *Enterococcus* spp and a central line was present in all cases. In 52/116 (45%) cases there was growth of another organism, and 61/116 (53%) cases had an alternative infectious cause for PNA, whether it was a typical respiratory bacterial pathogen, viral illness, or invasive fungal infection. In 55 (47%) cases, there was no alternative infectious cause of PNA identified; 44 (80%) of these patients received an antibiotic active against *Enterococcus* spp and of these, 30 (68%) were clinically improved after 14 days. Overall mortality was high with 52/116 total cases (45%) in which the patient was alive after 90 days. In 25 cases patients did not receive antibiotic therapy active against *Enterococcus* and 28% were alive at 90 days.

**Conclusion:**

*Enterococcus* spp should be considered a significant pulmonary pathogen in immunocompromised hosts in the appropriate clinical context. At our institution, the majority of immunocompromised patients with a monomicrobial pulmonary infection with *Enterococcus* were treated with targeted antibiotics. Further studies should evaluate outcomes of and indications for targeted treatment of pleuropulmonary *Enterococcus*.

**Disclosures:**

**All Authors**: No reported disclosures.

